# Loss of *porin* function in dopaminergic neurons of Drosophila is suppressed by *Buffy*

**DOI:** 10.1186/s12929-016-0300-1

**Published:** 2016-11-24

**Authors:** P. Githure M’Angale, Brian E. Staveley

**Affiliations:** Department of Biology, Memorial University of Newfoundland, St. John’s, Newfoundland & Labrador A1B 3X9 Canada

**Keywords:** α-synuclein, Buffy, Dopaminergic neurons, Mitochondria, Porin, Parkinson disease

## Abstract

**Background:**

Mitochondrial porin, also known as the voltage-dependent anion channel (VDAC), is a multi-functional channel protein that shuttles metabolites between the mitochondria and the cytosol and implicated in cellular life and death decisions. The inhibition of *porin* under the control of neuronal *Ddc-Gal4* result in short lifespan and in an age-dependent loss in locomotor function, phenotypes that are strongly associated with Drosophila models of Parkinson disease.

**Methods:**

Loss of *porin* function was achieved through exploitation of RNA interference while derivative lines were generated by homologous recombination and tested by PCR. The *UAS/Gal4* expression system was exploited with directed expression in neurons achieved with the use of the *Dopa decarboxylase* and in the developing eye with the *Glass multiple reporter* transgenes. Statistical analyses for ageing assay employed Log rank (Mantel-Cox) test, climbing indices were fitted with a non-linear curve and confidence intervals compared at 95%. Biometric analysis of the eye phenotypes was obtained by unpaired student *T*-test.

**Results:**

The expression of *α-synuclein* in neuronal populations that include dopamine producing neurons under the control of *Ddc-Gal4* produces a robust Parkinson disease model, and results in severely reduced lifespan and locomotor dysfunction. In addition, the *porin*-induced phenotypes are greatly suppressed when the pro-survival *Bcl-2* homologue *Buffy* is overexpressed in these neurons and in the developing eye adding to the cellular advantages of altered expression of this anti-apoptotic gene. When we co-expressed *α-synuclein* along with *porin*, it results in a decrease in lifespan and impaired climbing ability. This enhancement of the *α-synuclein-*induced phenotypes observed in neurons was demonstrated in the neuron rich eye, where the simultaneous co-expression of *porin-RNAi* and *α-synuclein* resulted in an enhanced eye phenotype, marked by reduced number of ommatidia and increased disarray of the ommatidia.

**Conclusions:**

The inhibition of *porin* in dopaminergic neurons among others result in reduced lifespan and age-dependent loss in climbing ability, phenotypes that are suppressed by the overexpression of the sole pro-survival *Bcl-2* homologue *Buffy*. The inhibition of *porin* phenocopies Parkinson disease phenotypes in Drosophila, while the overexpression of *Buffy* can counteract these phenotypes to improve the overall “healthspan” of the organism.

## Background

The voltage-dependent anion channel (VDAC), also known as mitochondrial porin, consists of small pore-forming proteins present in the outer mitochondrial membrane that act to shuttle nucleotides, metabolites and ions between the mitochondria and the cytoplasm [1, 2]. Porin is a multi-functional protein and is involved in the regulation of metabolism and energetic functions of the mitochondria and a constituent of the mitochondrial permeability transition pore (PTP) [3]. Porin is involved in apoptosis, metabolite transport, calcium transport and signalling, ATP transport, reactive oxygen species transport and endoplasmic reticulum – mitochondrial crosstalk [3–5]. As thus porin appears to be a convergence point for cell death and survival signals, mediated by its association with a variety of ligands and proteins. Porin is implicated in mitochondria-mediated apoptosis and in regulation of apoptosis through interaction with pro-survival proteins [3]. It interacts with the pro-survival hexokinase to mediate its anti-apoptotic activity [3, 6], and the Bcl-2 family of proteins to regulate mitochondria-mediated apoptosis [7, 8]. This association can induce cell survival or death.

The *porin* gene is associated with several neurodegenerative disorders including Alzheimer disease [9], Down syndrome [10], and dopamine-induced apoptosis [11]. The association of porin with Parkinson disease-associated gene products has been established, where it recruits parkin to defective mitochondria to promote mitophagy [12], and shows high affinity interaction with α-synuclein to regulate mitochondrial-induced toxicity [13]. This study suggests that α-synuclein translocate to the mitochondria via porin to target complexes of the mitochondrial respiratory chain. The accumulation and aggregation of abnormal α-synuclein was shown to down-regulate porin [14] and possibly regulate mitochondrial permeability [15]. The association between the PD gene *α-synuclein* and the mitochondrial channel *porin* appears to be important in the progression of PD. The initial Drosophila PD model employed the expression of human *α-synuclein* transgene to generate the PD-like phenotypes [16], that are commonly known as the *α-synuclein-*induced phenotypes. The success of this model anchors on its ability to phenocopy features of human PD such as the age-dependent loss in locomotor function and therefore, has found application in the study of *α-synuclein*-induced degeneration [16–23]. The use of the bipartite *UAS/GAL4* expression system [24], and the remarkable number of promoters or enhancers available, of which *TH-Gal4*, *elav-Gal4* and *Ddc-Gal4* are utilized in modelling PD in flies [16–23], makes Drosophila a useful and albeit a powerful model organism.

The loss of function of Drosophila *porin/VDAC* has been shown to result in mitochondrial morphological defects [25, 26]. These mitochondrial defects were accompanied by locomotor dysfunction and male sterility. In addition, *porin* mutants displayed neurological and muscular defects, mitochondrial respiratory defects, and abnormalities in synaptic transmission and mitochondrial distribution in motor neurons. Here we suppressed *porin* by RNA interference in Drosophila neurons under the control of the *dopa decarboxylase* transgene and analysed longevity and locomotor ability. Further we co-expressed *porin-RNAi* with *α-synuclein* to investigate its effects in the well-studied Drosophila PD model. The association of porin with Bcl-2 members is well documented, we have demonstrated the benefits of overexpression of the sole anti-apoptotic *Bcl-2* member *Buffy* in conditions of stress [27, 28], as thus, we overexpressed *Buffy* along with *porin-RNAi*. In addition, we altered the expression of *porin* in the Drosophila developing eye and co-expressed with *α-synuclein* and *Buffy*.

## Methods

### Bioinformatic analysis

The protein sequences were sourced from National Center for Biotechnology Information (NCBI; http://www.ncbi.nlm.nih.gov/protein/) while conserved domains were identified using the NCBI Conserved Domain Database (CDD; http://www.ncbi.nlm.nih.gov/cdd) [29] and the Eukaryotic Linear Motif [30] (http://elm.eu.org/) which focuses on annotation and detection of eukaryotic linear motifs (ELMs) or short linear motifs (SLiMs). Clustal Omega multiple sequence alignment (http://www.ebi.ac.uk/Tools/msa/clustalo/) [31, 32] was used to show conservation of the porin3_VDAC domain in the selected organisms. The nuclear export signal (NES) was predicted by NetNES (http://www.cbs.dtu.dk/services/NetNES/) [33] and TMpred, a program that predicts membrane-spanning regions and their orientation. The algorithm is based on the statistical analysis of TMbase, a database of naturally occurring transmembrane proteins (http://www.ch.embnet.org/software/TMPRED_form.html).

### Drosophila media and culture

Stocks and crosses were maintained on standard cornmeal/molasses/yeast/agar media treated with propionic acid and methylparaben to inhibit fungal growth. Stocks were maintained on solid media for 2 to 3 weeks before transfer onto new media to re-culture. Stocks were kept at room temperature (22 °C ± 2 °C) while crosses and experiments were carried out at 25 and 29 °C.

### Drosophila stocks

The *P{KK107645}VIE-260B* hereby referred to as *UAS-porin-RNAi (1)* was obtained from Vienna Drosophila Resource Center, *y[1] v[1]; P{y[+t7.7] v[+t1.8] = TRiP.JF03251}attP2/TM3, Sb[1]* hereby known as *UAS-porin-RNAi (2)*. Porin expression patterns are detailed in FlyBase http://flybase.org/reports/FBgn0004363.html, and in the Berkeley Drosophila Genome Project (BDGP; http://flybase.org/reports/FBgn0004363.html) [34]. Similarly, a thorough expression study was performed by Olivia et al., 2002 and showed a wide range of patterns [35]. *GMR-Gal4* [36] and *UAS-lacZ* flies were obtained from the Bloomington Drosophila Stock Center at Indiana University. *UAS-α-synuclein* [16] was generously provided by Dr. M. Feany of Harvard Medical School, *Ddc-Gal4* [37] by Dr. J. Hirsch of University of Virginia and *UAS-Buffy* [38] by Dr. L. Quinn of University of Melbourne. Studies to establish the expression pattern of *Buffy* have previously been performed [38, 39]. They detected *Buffy* mRNA via RT-PCR at all developmental stages, with the strongest expression being at the late larval/ early pupal stage [38]. The expression patterns correlate with regions of cell death and occurs in the same pattern as the pro-cell death *Debcl* [38, 40]. Additional expression data is found on FlyBase http://flybase.org/reports/FBgn0040491.html.

### Drosophila derivative lines

The *UAS-α-synuclein/CyO; Ddc-Gal4/TM3, UAS-α-synuclein/CyO; GMR-Gal4, UAS-Buffy/CyO; Ddc-Gal4* and *UAS-Buffy/CyO; GMR-Gal4* derivative lines were generated using standard homologous recombination methods that we have previously described [41, 42] and were used for the overexpression of either *α-synuclein* or *Buffy* in DA and other neurons using the *Ddc-Gal4* transgene or in the developing eye using the *GMR-Gal4* transgene. PCR reaction was used to determine the amplification of DNA products and Gel electrophoresis was used for confirmation of recombination events via presence of the PCR product.

### Ageing assay

The analysis for survival was performed following a protocol that has previously been described [27, 43]. But briefly, from each genotype crosses were made and a cohort of at least two hundred flies collected and aged. Flies were considered dead when they did not display movement upon agitation [44]. Survival curves were compared using the log-rank (Mantel-Cox) test and significance was determined at 95%, at a *P*-value less than or equal to 0.05 with Bonferroni correction.

### Climbing assay

Analysis for climbing ability was determined using a standard protocol that was described in our laboratory [45]. This assay scores the flies ability to climb over their lifetime and analyses 50 males from every genotype. Climbing indices obtained were analysed using GraphPad Prism version 5.04 and climbing curves were fitted using non-linear regression. Comparisons were done at a 95% confidence interval with a *P*-value threshold of less than 0.05 considered significant.

### Scanning electron microscopy of the Drosophila eye

The Drosophila eyes for scanning electron microscopy and analysis were prepared following a standard protocol, as previously described [27]. At least 10 different eye images per genotype were analysed using the National Institutes of Health (NIH) ImageJ software [46]. The proportion of the disrupted eye area was calculated as detailed in a previous publication [47]. Statistical comparisons were evaluated using a one-way analysis of variance followed by a Dunnett’s multiple comparison tests. *P*-values less than 0.05 were considered significant.

## Results

### The human and Drosophila porin domain is conserved

**Fig. 1:**
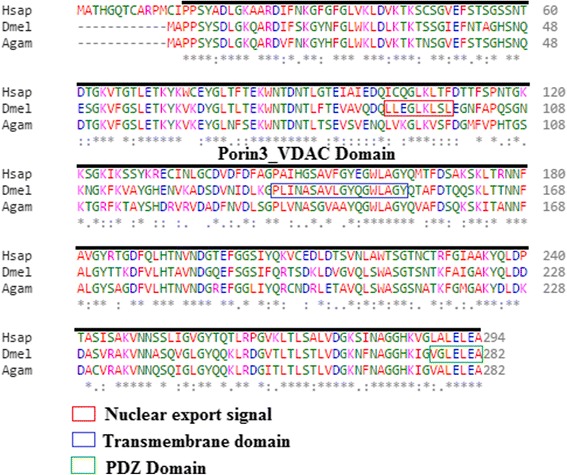
Drosophila porin has a conserved Porin3_VDAC domain. The *Drosophila melanogaster porin* gene encodes a 282 amino acids protein and the Porin domain is highly conserved when compared to the human homologue. It shows presence of a nuclear export signal (NES), a transmembrane domain, and a PDZ domain. Domains were identified using the NCBI Conserved Domain Database Search (CDD) [29] and the Eukaryotic Linear Motif resource search [30]. A Clustal Omega multiple sequence alignment [31, 32] show conservation of the porin3_VDAC domain (Hsap is *Homo sapiens* NP_003366.2, Dmel is *Drosophila melanogaster* NP_001033899.1 and Agam is *Anopheles gambiae* XP_318947.2). “*” indicate the residues that are identical, “:” indicate the conserved substitutions, “.” indicate the semi-conserved substitutions. Colours show the chemical nature of amino acids. Red is small hydrophobic (including aromatic), Blue is acidic, Magenta is basic, and Green is basic with hydroxyl or amine groups

There is 62% identity and 77% similarity between the human porin (VDAC) and the *Drosophila melanogaster* porin protein sequences, with very high conservation within the Porin3_VDAC domain (Fig. 1). The putative dimerization interface and putative determinants of voltage-gated binding sites are well conserved as determined by an NCBI conserved domain search [29]. A Eukaryotic linear motif (ELM) resource search for functional sites [48] in the Drosophila transcript indicates the presence of an inhibitor of apoptosis binding motif (IBM) that function in the abrogation of caspase inhibition by inhibitors of apoptosis (IAPs) at amino acids 1 to 5, an Atg8 binding motif at amino acids 5 to 9, a nuclear export signal (NES) at amino acids 91 to 98, a PDZ domain at amino acids 277 to 282 and a transmembrane domain predicted by TMpred.

### Inhibition of *porin* in neurons decreases lifespan and severely impairs locomotor function, phenotypes that are suppressed by *Buffy* overexpression

**Fig. 2:**
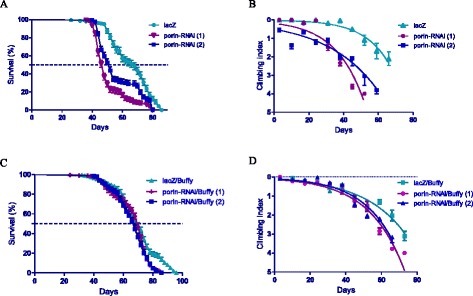
Loss of porin activity decreases survival and impairs climbing ability. **a** The inhibition of *porin* in neurons using the *Ddc-Gal4* transgene results in decreased median lifespan of 48 and 52 days when compared to 68 days for control flies that expresses *UAS-lacZ*. The genotypes are *Ddc-Gal4/ UAS-lacZ*, *Ddc-Gal4/ UAS-porin-RNAi (1)* and *Ddc-Gal4/ UAS-porin-RNAi (2).* Longevity is shown as percent survival (*P* < 0.05, determined by the log-rank (Mantel-Cox) test and *n* ≥ 200). **b** The inhibition of *porin* in the *Ddc-Gal4*-expressing neurons resulted in a significant decrease in climbing ability as determined by nonlinear fitting of the climbing curves and comparing 95% CI. The genotypes are *Ddc-Gal4/ UAS-lacZ*, *Ddc-Gal4/ UAS-porin-RNAi (1)* and *Ddc-Gal4/ UAS-porin-RNAi (2).* Error bars indicate SEM and *n* = 50. **c** The co-expression of *Buffy* with *porin-RNAi* result in the rescue of the observed phenotype of decreased survival, with a median survival of 70 and 69 days when compared to 72 days for controls. Genotypes are *Ddc-Gal4 UAS-Buffy/ UAS-lacZ*, *Ddc-Gal4 UAS-Buffy/ UAS-porin-RNAi (1)* and *Ddc-Gal4 UAS-Buffy/ UAS-porin-RNAi (2).* Longevity is shown as percent survival (*P* < 0.05, determined by log-rank (Mantel-Cox) test with *n* ≤ 200). **d** The inhibition of *porin* along with the overexpression of *Buffy* in the DA neurons results in the suppression of the age-dependent loss in climbing ability. The genotypes are *Ddc-Gal4 UAS-Buffy/ UAS-lacZ*, *Ddc-Gal4 UAS-Buffy/ UAS-porin-RNAi (1)* and *Ddc-Gal4 UAS-Buffy/ UAS-porin-RNAi (2).* Analysis was done by nonlinear fitting of the climbing curves and significance was determined by comparing the 95% CI. Error bars indicate SEM and *n* = 50

The expression of *porin-RNAi* in *Ddc-Gal4*-expressing neurons results in a slightly decreased lifespan and severely impaired locomotor function as shown by the two RNAi lines that we tested. The median lifespan for these flies was 48 and 52 days when compared to 68 days for the controls as determined by Log-rank (Mantel-Cox) test with a *p* < 0.0001 (Fig. 2a). When *porin* is suppressed in these neurons, the flies have impaired locomotor ability as determined by comparison of CI after the nonlinear fit of the climbing curves (Fig. 2b). These results suggest a role for *porin* in the normal function of neurons in Drosophila since its reduced activity shortens lifespan and prematurely retards climbing ability.

The directed overexpression of the pro-survival *Bcl-2* homologue *Buffy* in these neurons resulted in increased lifespan and improved climbing ability. When *Buffy* is co-expressed with the *porin-RNAi* lines, the results indicate a median lifespan of 70 and 69 days when compared to 72 days for *Buffy* co-expression with *lacZ* control flies as determined by Log-rank test (Fig. 2c). The climbing ability of the *porin-RNAi* flies was significantly improved as determined by comparison of climbing curves of *porin-RNAi* flies at 95% CI (Fig. 2b) with the flies that express *porin-RNAi* along with *Buffy* and with the control flies that co-expressed *Buffy* along with *lacZ* (Fig. 2d). Taken together these results suggest a pro-survival role for *Buffy* as observed by significant increases in the “healthspan” of *porin*-deficient flies.

### Inhibition of *porin* enhances *α-synuclein-*dependent phenotypes

**Fig. 3:**
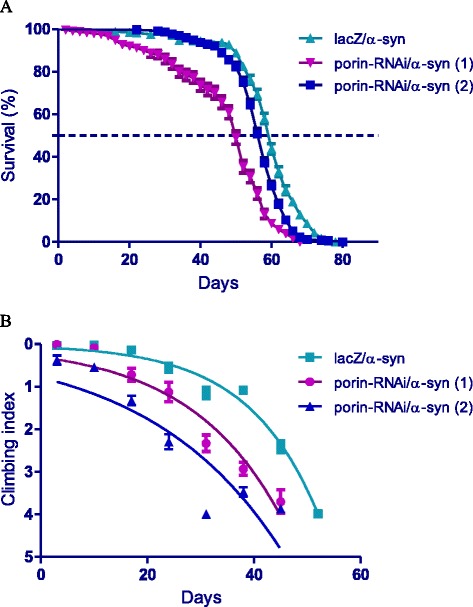
Loss of *porin* function enhances the *α-synuclein-*induced reduction in lifespan and age-dependent loss of climbing ability. **a** The directed inhibition of *porin* along with *α-synuclein* expression in the neurons decreased lifespan with a median survival of 50 and 56 days when compared to 60 days for the control flies that express *α-synuclein* along with the *lacZ* transgene. Genotypes are *UAS-α-synuclein; Ddc-Gal4/ UAS-lacZ*, *UAS-α-synuclein; Ddc-Gal4/UAS-porin-RNAi (1)* and *UAS-α-synuclein; Ddc-Gal4/ UAS-porin-RNAi (2).* Longevity is shown as percent survival (*P* < 0.05, determined by log-rank (Mantel-Cox) test with *n* ≤ 200). **b** The co-expression of *porin-RNAi* with *α-synuclein* resulted in reduction of climbing ability over time when compared to the controls. The genotypes are *UAS-α-synuclein; Ddc-Gal4/UAS-lacZ*, *UAS-α-synuclein; Ddc-Gal4/UAS-porin-RNAi (1)* and *UAS-α-synuclein; Ddc-Gal4/UAS-porin-RNAi (2).* Analysis was done by nonlinear fitting of the climbing curves and significance was determined by comparing the 95% CI. Error bars indicate SEM and *n* = 50

The expression of *α-synuclein* in *Ddc-Gal4*-expressing neurons results in impaired locomotor function that has been attributed to cellular toxicity due to the accumulation of this protein. The co-expression of the *porin-RNAi* lines along with *α-synuclein*, decreased survival and impaired climbing ability over time (Fig. 3). The median lifespan was 50 and 56 days for flies that expressed *porin-RNAi* along with *α-synuclein,* compared to 60 days for control flies that co-expressed *α-synuclein* along with *lacZ*, a significant decrease in survival for both RNAi lines (Fig. 3a) as determined by Log-rank (Mantel-Cox) test (*p* < 0.0001). A comparison of the climbing curves by nonlinear fitting at 95% CI revealed they were significantly different (Fig. 3b), with CI of 0.04691 to 0.06795 and 0.030 to 0.050 for flies that expressed *porin-RNAi* along with *α-synuclein,* compared to 0.06842 to 0.08366 for control flies that co-expressed *α-synuclein* along with *lacZ*. This suggests that the inhibition of *porin* together with the expression of *α-synuclein* in these neurons confers a significant health disadvantage, with marked decreases in survival and premature loss of climbing ability.

### Inhibition of *porin* in the eye decreases ommatidia number and increases ommatidial disarray, phenotypes that are rescued by *Buffy* overexpression

**Fig. 4:**
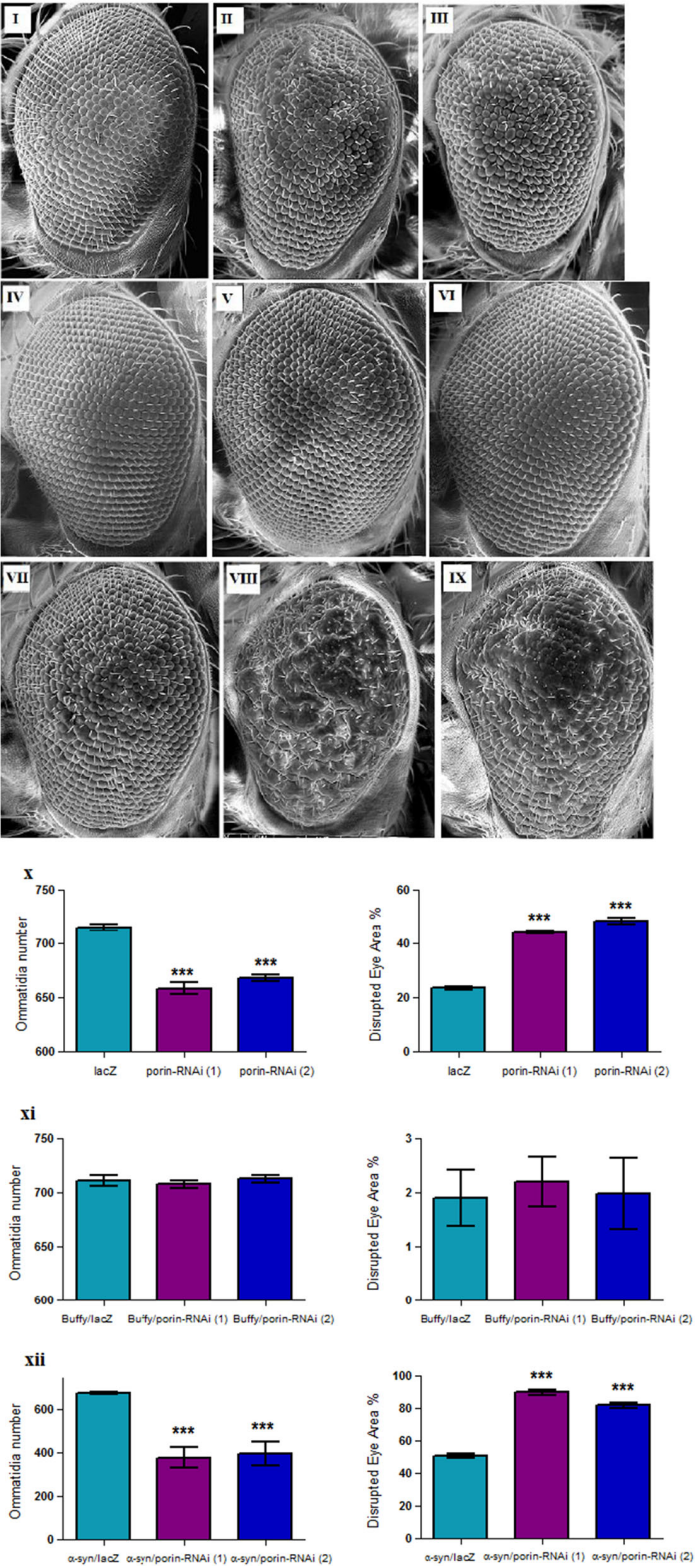
Inhibition of *porin* in the developing eye results in phenotypes that may be suppressed by *Buffy* and enhanced by *α-synuclein.* Scanning electron micrographs when *porin* is inhibited in the developing eye and co-expressed along with either *Buffy* or *α-synuclein.* The genotypes are (**i**) *GMR-Gal4/ UAS-lacZ,* (**ii**) *GMR-Gal4/ UAS-porin-RNAi (1),* (**iii**) *GMR-Gal4/ UAS-porin-RNAi (2),* (**iv**) *UAS-Buffy; GMR-Gal4/ UAS-lacZ,* (**v**) *UAS-Buffy; GMR-Gal4/ UAS-porin-RNAi (1),* (**vi**) *UAS-Buffy; GMR-Gal4/UAS-porin-RNAi (2),* (**vii**) *UAS-α-synuclein; GMR-Gal4/UAS-lacZ,* (**viii**) *UAS-α-synuclein; GMR-Gal4/ UAS-porin-RNAi (1),* and (**ix**) *UAS-α-synuclein; GMR-Gal4/UAS-porin-RNAi (2)*. Biometric analysis when (**x**) *porin* is inhibited in the eye indicated decreased ommatidia number and higher percentage of ommatidial disruption when compared to the control. (**xi**) The overexpression of *Buffy* with *porin-RNAi* results in restoration of the number of ommatidia and the degree of ommatidial disruption to below the control levels. (**xii**) The inhibition of *porin* along with *α-synuclein* expression resulted in the enhancement of the eye phenotypes when compared to controls as displayed by the low number of ommatidia coupled by the high degree of disruption of the ommatidial array. All comparisons were determined by a one-way analysis of variance followed by a Dunnett’s multiple comparison test (*P* < 0.05), error bars are SEM, asterisks (*) represent statistical significance and *n* = 10

When *porin-RNAi* is directed in the developing eye using the *GMR-Gal4* transgene, it results in eyes with decreased number of ommatidia and higher disruption of the ommatidial array (Fig. 4ii, iii and x) as determined by a one-way analysis of variance followed by a Dunnett’s multiple comparison test *p* < 0.0001. Co-expression of *porin* with *Buffy* restored the mean number of ommatidia and the percentage disruption to control levels as determined by a one-way analysis of variance followed by a Dunnett’s multiple comparison test *p* > 0.05 (Fig. 4v, vi and xi). Taken together, these results suggest that *porin* may play a role in the development of the Drosophila eye and that *Buffy* suppresses the developmental eye defects that result from the inhibition of *porin*. The inhibition of *porin* along with *α-synuclein* overexpression resulted in a significant decrease in the number of ommatidia due to fusion of ommatidia and an increase in the percentage disruption of the eye (Fig. 4 viii, ix and xii) as determined by a one-way analysis of variance followed by a Dunnett’s multiple comparison test *p* < 0.0001. This suggests an enhancement of the neurotoxic effects of the *α-synuclein-*induced developmental eye defects in the presence of reduced *porin* activity.

## Discussion

The multitude of functions attributed to mitochondrial porin or VDAC and its control of the entry and exit of mitochondrial metabolites makes it a key player in the cellular decisions that lead to either survival or death [1]. The expression of *porin-RNAi* in neurons under the direction of the *Ddc-Gal4* transgene results in shortened lifespan and a premature loss in locomotor ability, results that were consistent in both RNAi lines tested and that corroborate previous studies [25, 26]. This gene product is involved in maintaining mitochondrial morphology, and its disruption leads to a host of phenotypes among them locomotor defects. In our study, we disrupted this protein in DA and other neurons, the results obtained signifies a close connection between *porin* and the progression of the PD-like phenotypes of shortened lifespan and an age-dependent loss in locomotor function. The comparison of climbing indices of flies at 40 days when most of them are alive to the control lines indicate a significant change in the phenotypes, these appears to be a strong indication of possible neurodegeneration.

The relationship between mitochondrial porin and PD susceptibility gene products has been investigated in other organisms [12–14, 49, 50]. The inhibition of *porin* along with the expression of *α-synuclein* in *Ddc-Gal4*-expressing neurons of *Drosophila melanogaster* results in the enhancement of the loss of *α-synuclein*-induced phenotypes, with a decrease in lifespan and an impairment in climbing ability. Some studies have attributed the neurotoxicity of *α-synuclein* to its interaction with electron transport chain components among them Complex I [51]. It has been suggested that α-synuclein blocks the activity of porin and uses this channel to translocate into the inner mitochondria [13] and that it preferentially interacts with mitochondrial membranes compared to other organelle membranes [52]. This association inhibits mitochondrial function and promotes reactive oxygen stress. Our study firstly inhibited the mitochondria *porin* and secondly expressed *α-synuclein* in the same neurons, this resulted in the enhancement of the observed phenotypes, with shortened lifespan and severe reduction in climbing ability over time. It seems therefore that the combination effect of the directed inhibition of *porin*, and expression of *α-synuclein* confers a greater disadvantage to “healthspan”, albeit when altered in neurons. When altered individually, *α-synuclein-*induced PD model, a well-studied and robust disease model in Drosophila [16, 22] result in shortened lifespan and impaired climbing ability. Inhibition of *porin* in the developing eye results in extensive ommatidial disruption and fewer ommatidia number, because of intensive fusion of the ommatidia with no distinct ommatidia detectable in most of the eyes analysed. We suggest that though α-synuclein interacts with the mitochondria to result in disruption of mitochondria homeostasis, loss of *porin* in neurons seem to be independent of *α-synuclein-*induced phenotypes and this highlights the complexity of mechanisms involved in the pathogenesis of PD.

The association of porin with members of the Bcl-2 family is well documented [7], and has been suggested to be a point of convergence for cell survival and death signals [3]. When we overexpressed *Buffy*, the sole pro-survival *Bcl-2* homologue, in Drosophila neurons, along with inhibition of *porin* via RNAi, the phenotypes associated with the loss-of-function of *porin*, shortened lifespan and impaired climbing ability, were suppressed. The survival-induced advantages of Buffy especially under conditions of stress are well documented [27, 28, 38, 41, 42], and so is the regulation of porin by Bcl-2 proteins that underscores the importance of Bcl-2 protein in life and death decisions. The overexpression of *Buffy* along with the inhibition of *porin* in *Ddc-Gal4*-expressing neurons and in the developing eye resulted in a suppression of the phenotypes. The excess Buffy product must therefore confer cellular advantages to the target cells and counteracts the toxic effects of *porin* inhibition, and demonstrates a wider role for the Drosophila pro-survival homologue, with potential involvement in the mitochondria-mediated cell death. The developmental expression patterns of *Buffy* and *porin* can shed light on the resulting phenotypes and possibly on the counteraction of the *porin*-induced phenotypes by overexpression of *Buffy*. One study has suggested that *porin* was not involved in *debcl*-induced cell death [25] and found that apoptosis induced by *debcl* overexpression was not inhibited by *porin* loss of function. As such it seems that the rescue of *porin*-induced phenotypes by *Buffy* are consistent with its action on the mitochondria directly or through other proteins in a dedicated pro-survival signalling pathway.

## Conclusions

The inhibition of *porin* in the *Ddc-Gal4*-expressing neurons and the developing eye is rescued upon the overexpression of *Buffy*, a pro-survival *Bcl-2* homologue. The co-expression of *porin-RNAi* along with *α-synuclein* results in enhanced phenotypes, this highlights the complexity of *α-synuclein-*induced mechanisms in the pathogenesis of PD, and in deed demonstrates the multi-faceted mechanisms involved in the aetiology of PD.

## Abbreviations

Bcl-2: B cell lymphoma 2
CI: Confidence interval
DA: Dopaminergic
Ddc: DOPA decarboxylase
GMR: Glass Multimer Reporter
RNAi: Ribonucleic acid interference
SEM: Standard error of the mean
VDAC: Voltage-dependent anion channel
